# Efficacy and safety of sodium-glucose cotransporter-2 inhibitors for heart failure with mildly reduced or preserved ejection fraction: a systematic review and meta-analysis of randomized controlled trials

**DOI:** 10.3389/fcvm.2023.1273781

**Published:** 2023-10-12

**Authors:** Huzaifa Ahmad Cheema, Arman Shafiee, Mohammad Mobin Teymouri Athar, Mohammad Ali Rafiei, Atefe Mehmannavaz, Kyana Jafarabady, Abia Shahid, Adeel Ahmad, Sardar Hassan Ijaz, Sourbha S. Dani, Abdul Mannan Khan Minhas, Abdulqadir J. Nashwan, Marat Fudim, Gregg C. Fonarow

**Affiliations:** ^1^ Department of Cardiology, King Edward Medical University, Lahore, Pakistan; ^2^ Clinical Research Development Unit, Alborz University of Medical Sciences, Karaj, Iran; ^3^ Student Research Committee, School of Medicine, Alborz University of Medical Sciences, Karaj, Iran; ^4^ School of Medicine, Shahid Beheshti University of Medical Sciences, Tehran, Iran; ^5^ Department of Internal Medicine, Mass General Brigham - Salem Hospital, Salem, MA, United States; ^6^ Lahey Hospital and Medical Center, Burlington, MA, United States; ^7^ Department of Medicine, University of Mississippi Medical Center, Jackson, MS, United States; ^8^ Hamad Medical Corporation, Doha, Qatar; ^9^ Department of Medicine, Duke University Medical Center, Durham, NC, United States; ^10^ Duke Clinical Research Institute, Durham, NC, United States; ^11^ Ahmanson-UCLA Cardiomyopathy Center, Division of Cardiology, University of California Los Angeles, Los Angeles, CA, United States

**Keywords:** SGLT2I, empagliflozin, dapagliflozin, heart failure, HFPEF

## Abstract

**Aims:**

We sought to conduct a meta-analysis to evaluate the efficacy and safety of sodium-glucose cotransporter-2 inhibitors (SGLT2i) in patients with heart failure (HF) with preserved ejection fraction (HFpEF) and HF with mildly reduced ejection fraction (HFmrEF).

**Methods:**

We searched the Cochrane Library, MEDLINE (via PubMed), Embase, and ClinicalTrials.gov till March 2023 to retrieve all randomized controlled trials of SGLT2i in patients with HFpEF or HFmrEF. Risk ratios (RRs) and standardized mean differences (SMDs) with their 95% conﬁdence intervals (95% CIs) were pooled using a random-effects model.

**Results:**

We included data from 14 RCTs. SGLT2i reduced the risk of the primary composite endpoint of first HF hospitalization or cardiovascular death (RR 0.81, 95% CI: 0.76, 0.87; *I*^2^ = 0%); these results were consistent across the cohorts of HFmrEF and HFpEF patients. There was no significant decrease in the risk of cardiovascular death (RR 0.96, 95% CI: 0.82, 1.13; *I*^2^ = 36%) and all-cause mortality (RR 0.97, 95% CI: 0.89, 1.05; *I*^2^ = 0%). There was a significant improvement in the quality of life in the SGLT2i group (SMD 0.13, 95% CI: 0.06, 0.20; *I*^2^ = 51%).

**Conclusion:**

The use of SGLT2i is associated with a lower risk of the primary composite outcome and a higher quality of life among HFpEF/HFmrEF patients. However, further research involving more extended follow-up periods is required to draw a comprehensive conclusion.

**Systematic Review Registration:**

PROSPERO (CRD42022364223).

## Introduction

Heart failure (HF) is a complex clinical syndrome that results from the impaired ability of the ventricle to fill or eject blood (1). Based on ejection fraction (EF), HF is categorized as HF with preserved ejection fraction (HFpEF): EF ≥50%, HF with mildly reduced ejection fraction (HFmrEF): EF of 41%–49%, and HF with reduced ejection fraction (HFrEF): EF <40% (2). Nearly half of the patients with a diagnosis of HF have a normal or near-normal EF (3). HFpEF is a major global public health concern causing substantial morbidity and mortality (4, 5). With the ageing population and increasing prevalence of comorbidities, the prevalence of HFpEF and HFmrEF is estimated to increase (2, 6).

The sodium-glucose cotransporter-2 inhibitors (SGLT2i) have been established as an important component in the management of HFrEF; however, they have a weaker (class 2a) recommendation in the 2022 American College of Cardiology (ACC)/American Heart Association (AHA) guidelines for HFmrEF and HFpEF (7) although in the most recent update of the European guidelines they have gotten a class Ia recommendation for reducing the risk of HF hospitalization or cardiovascular death (8). There is a growing body of literature establishing the efficacy and benefits of these drugs in patients with HFmrEF and HFpEF. The results of the DELIVER trial, the largest trial to date regarding the use of SGLT2i in HFpEF/HFmrEF patients, have recently been published (9). Furthermore, the results of the individual randomized controlled trials (RCTs) are underpowered in some outcomes such as cardiovascular mortality (9). Therefore, we aimed to conduct this systematic review and meta-analysis to evaluate the safety and efficacy of SGLT2i for managing patients with HFpEF and HFmrEF to inform clinical decision-making.

## Methods

We conducted our meta-analysis in conformity with the Cochrane Handbook for Systematic Reviews of Intervention (10) and The Preferred Reporting Items for Systematic Reviews and Meta-Analyses (PRISMA) guidelines (11) (Supplementary Table 1). In addition, we prospectively registered our protocol with PROSPERO (CRD42022364223).

### Search strategy

We searched the following databases and registries from their inception to March 2023: Cochrane Central Register of Controlled Trials (CENTRAL, via The Cochrane Library), MEDLINE (via PubMed), Embase, and ClinicalTrials.gov. The search was conducted using different combinations of the following keywords: (“Sodium-Glucose Transporter 2 Inhibitors” OR “Canagliflozin” OR “dapagliflozin” OR “Empagliflozin” OR “Ipragliflozin” OR “Luseogliflozin” OR “Sotagliflozin” OR “Ertugliflozin”) AND (“Heart Failure”) (Supplementary Table 2). Our search also included bibliographies of identified articles.

### Study selection and data extraction

Inclusion criteria for eligible articles were defined as: (1) RCTs only; (2) patient population with HF with preserved/mildly reduced ejection fraction; (3) treatment with any SGLT2i vs. placebo or usual treatment. Trials that were conducted in diabetes mellitus patients but provided data as subgroup or secondary analyses on HFpEF/HFmrEF patients were also included. Meanwhile, observational studies, and animal studies were excluded.

Two reviewers performed the screening process in EndNote X9, including duplication removal, screening via titles and abstracts, and finally, full texts were examined. A third reviewer was asked to assess to decrease the screening bias and resolve the disagreements.

Data were obtained from text, tables, figures, and supplementary materials. Two reviewers independently extracted data and classified them as follows: information about study characteristics (trial name, author name, year of publication, country of the region, type of study), population [the total number of participants, intervention and control descriptions, age, gender, left ventricular ejection fraction (LVEF) and diabetes prevalence], and interventions (diagnostic threshold, duration, and dose of intervention).

### Quality assessment and certainty of evidence

Two reviewers independently assessed included studies using the revised Cochrane Risk of Bias tool (RoB 2.0). RoB 2.0 was used for the assessment of the following five domains: (1) selection bias, (2) performance bias, (3) detection bias, (4) attrition bias, and (5) reporting bias. Studies were classified into “high risk,” unclear risk,” and low risk based on the ROB-2 checklist.

To assess the certainty of evidence for each of our outcomes, the ﬁve Grades of Recommendation, Assessment, Development, and Evaluation (GRADE) considerations (study limitations, consistency of effect, imprecision, indirectness, and publication bias) were utilized to assess the certainty of the body of evidence (12).

### Outcomes

Primary outcomes included a composite endpoint of cardiovascular death or first HF hospitalization/urgent hospital visit due to heart failure, the incidence of cardiovascular death, and the risk of hospitalization. Secondary outcomes included risk of all-cause mortality, quality of life (using The Kansas City Cardiomyopathy Questionnaire (KCCQ) and Minnesota Living with Heart Failure (MLHF) scales), any adverse events (AEs), and serious adverse events (SAEs). Hypotension, hypoglycemia, ketoacidosis, drug discontinuation, and urinary tract infection were defined as specific adverse events of interest.

### Statistical analysis

We summarized the pooled effect size of dichotomous outcomes using the risk ratio (RR) and 95% conﬁdence intervals (95% CI). The standardized mean difference (SMD) was utilized for reporting the results of continuous outcomes. Study heterogeneity was assessed using the Chi-square test and *I*^2^ statistic. *P* < 0.10 was considered statistically signiﬁcant for the Chi-square Test. A DerSimonian and Laird random-effects approach was used in our meta-analysis. To investigate any potential effects of the individual moderators, subgroup analyses were carried out based on the diabetes status of patients, the type of study (whether an HFpEF-specific trial or a subgroup/post-hoc analysis), the EF diagnostic threshold used as inclusion criteria or cutoff for subgroup analysis by the studies (EF >40%, 45% or 50%), and the baseline LVEF of patients (40%-50% vs. >50%) for the primary outcomes. Publication bias was not assessed since there were fewer than 10 studies for all outcomes. All statistical analyses were carried out using RevMan version 5.4.

## Results

### Search results and study characteristics

**Figure 1:**
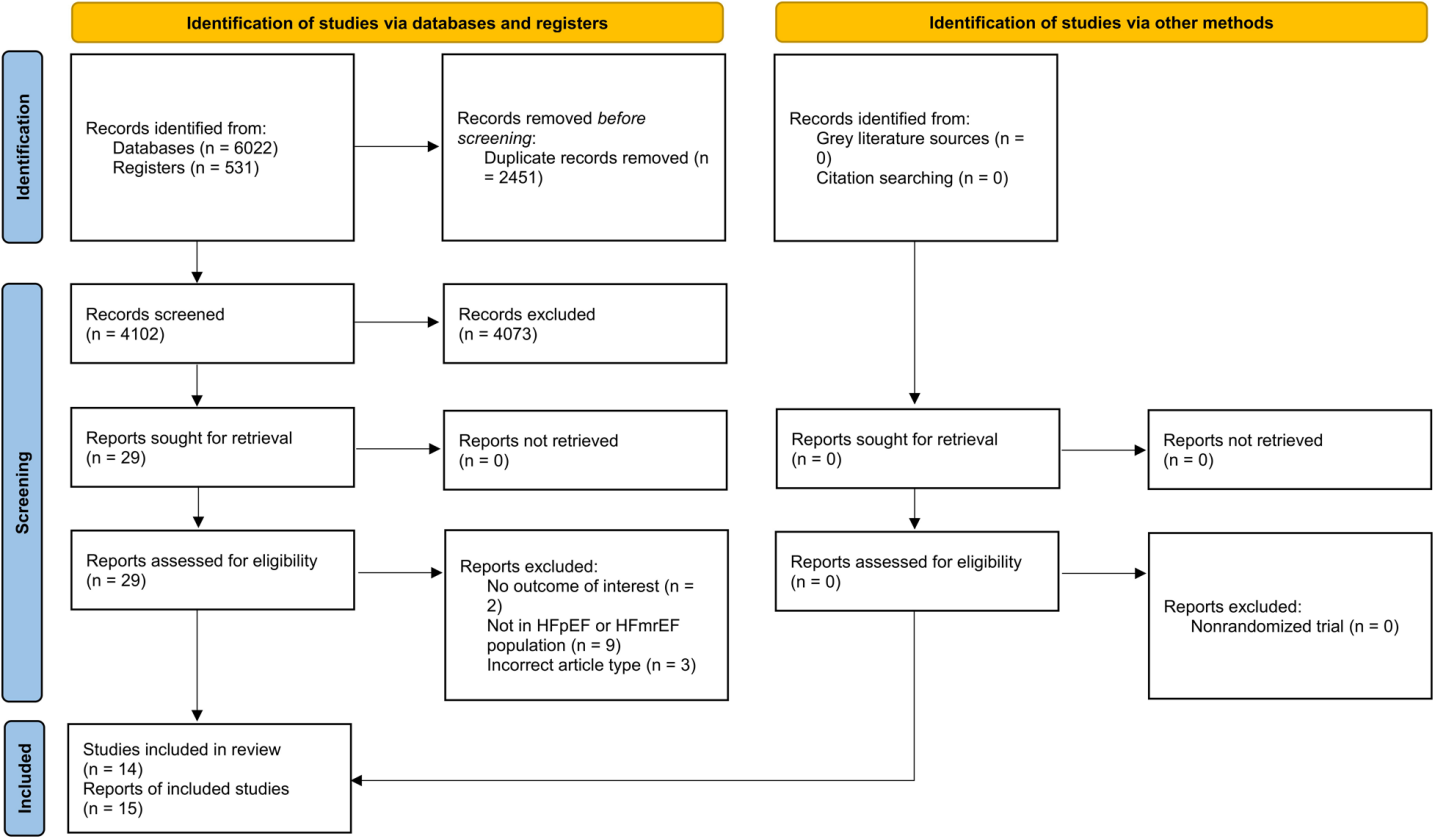
PRISMA 2020 flowchart of study selection process.

The detailed search result has been provided in Figure 1. Fifteen studies met the eligibility criteria and were included (9, 13, 22–26, 14–21). These reports provided data regarding 14 RCTs. Most of the studies were conducted in the USA. Among the included studies, eight were designed specifically for HFpEF patients (HFpEF-specific trial). For the definition of HFpEF/HFmrEF, five studies designated EF >40%, four designated EF >45%, and six designated EF >50% as the diagnostic thresholds to distinguish from HFrEF. The main population of 9 studies was diabetic patients. The duration of SGLT2i treatment ranged from 12 weeks to 3.5 years. Detailed characteristics of the included studies are available in Table 1.

**Table 1:** Characteristics of the included studies.

Study ID	Author, year	Location	Type of study	Sample size	Population	Diagnostic threshold of LVEF	Age	Male—no. (%)	NYHA class no. (%)	Baseline LVEF (mean/median)	Diabetes mellitus- no. (%)	Interventions	Follow-up duration	Initiation time-point	Other medications
EMPERIAL	Abraham William T., et al. (13)	USA	HFpEF-Specific trial	315 (157vs158)	HFrEF, HFpEF with and without T2D	HFrEF <_40% HFpEF >40%	HFpEF: 74.0 (68.0, 79.0) vs. 75.0 (68.0, 81.0)	87 (55.4) vs. 92 (58.2)	II:117 (74.5) vs. 126 (79.7) III:39 (24.8) vs. 32 (20.3)	53.0 (45.0, 58.0) vs. 53.0 (46.0, 59.0)	86 (54.8) vs. 75 (47.5)	empagliflozin 10 mg daily vs. placebo	12 weeks	at least 3 months after diagnosis of heart failure	On medical therapy for heart failure consistent with prevailing cardiovascular guidelines at a stable dose for ≥4 weeks prior to screening, except for diuretics which must have been stable for ≥2 weeks prior to screening. ACE inhibitors/ARBs, Beta-blockers, Mineralocorticoid receptor antagonist, Loop or high-ceiling diuretics, Thiazides or low ceiling diuretics, Lipid-lowering drugs
EMPEROR-Preserved	Anker Stefan D., et al. (15)	Germany	HFpEF-Specific trial	5,988 (2,997 vs. 2,991)	HFpEF	HFpEF >40%	71.8 ± 9.3 vs. 71.9 ± 9.6	1,659 (55.4) vs. 1,653 (55.3)	I:0.1 vs. <0.1 II:81.1vs 81.9 III:18.4 vs. 17.8 IV:0.3 vs. 0.3	54.3 ± 8.8 vs. 54.3 ± 8.8	1,466 (48.9) vs1472 (49.2)	empagliflozin10 mg daily vs. placebo	median: 26.2 months	at least 3 months after diagnosis of heart failure	usual therapy for HF (ARB, ACE inhibitors, ARNI, MRA) or other medical conditions
The SOLOIST-WHF trial	Bhatt D. L. et al. (16)	USA	Subgroup analysis	494	diabetics with HF	HFpEF >50%	N.R. separately for HFpEF or HFmrEF patients	N.R. separately for HFpEF or HFmrEF patients	N.R. separately for HFpEF or HFmrEF patients	N.R. separately for HFpEF or HFmrEF patients	all patients were diabetic	200 mg of sotagliflozin daily (with a dose increase to 400 mg, depending on side effects) VS placebo	median: 7.8 months	before or within 3 days after hospital discharge	no need for intravenous inotropic or vasodilator therapy (excluding nitrates), and having transitioned from intravenous to oral diuretic therapy, no treatment with IV inotropic or IV vasodilators within 2 days prior to Randomization Prior chronic treatment (or prescription) with a loop diuretic (e.g., furosemide, torsemide, bumetanide) for ≥30 days prior to the Index Event
SCORED	Bhatt D. L., et al. (17)	USA	Subgroup analysis	2248 (1,133 vs. 1,115)	HFmrEF or HFpEF T2D CKD Additional cardiovascular risk factors	HFpEF ≥50% 50%> HFrEF ≥40%	N.R. separately for HFpEF or HFmrEF patients	N.R. separately for HFpEF or HFmrEF patients	N.R. separately for HFpEF or HFmrEF patients	N.R. separately for HFpEF or HFmrEF patients	all patients were diabetic	sotagliflozin 200 mg once daily (400 mg once daily if unacceptable side effects did not occur) vs. placebo	sotagliflozin 14.2 months (10.3–18.9) vs. placebo 14.3 months (10.3–18.9)	NA	standard-of-care treatments (RAAS inhibitor, Calcium-channel blocker, diuretic, Metformin, Sulfonylurea, DPP-4 inhibitor, Insulin, GLP-1 receptor agonist
MUSCAT-HF trial	Ejiri K., et al. (19)	Japan	HFpEF-Specific trial	total (165) Luseogliflozin (83) vs. Voglibose (82)	Diabetics HFpEF	HFpEF ≥45%	71.7 ± 7.7 vs. 74.6 ± 7.7	55 (66) vs. 48 (59)	I:0 vs. 0 II:79 (96) vs. 81 (99) III:3 (4) vs. 1 (1) IV: 0 vs. 0	57 ± 9.4 vs. 58 ± 9.4	all patients were diabetic	luseogliflozin (2.5 mg once daily) vs. voglibose (0.2 mg 3 times daily)	12 weeks	NR	More than half of the patients in this study were treated with specific heart failure treatment drugs, such as angiotensin-converting enzyme inhibitors, angiotensin receptor blockers, and beta-blockers. Mineralocorticoid receptor antagonists were used in almost 20% of the patients. Antidiabetic medication was administered in, patients. ACE inhibitor or ARB, Beta-blocker, MRA, Loop diuretic, Hydralazine
The PRESERVED-HF trial	Nassif M. E., et al. (21)	USA	HFpEF-Specific trial	324 (162 vs. 162)	HFpEF	HFpEF ≥45%	69 (64, 77) VS 71 (63, 78)	70 (43.2) vs. 70 (43.2)	II:96 (59.3%) vs. 90 (55.6%) III/IV:65 (40.1%) vs. 72 (44.4%)	60 (55, 65) vs. 60 (54, 65)	90 (55.6%) vs. 91 (56.2%)	oral dapagliflozin 10 mg or matching placebo once daily	12 weeks	At least 7 days after hospital discharge and either HF hospitalization or urgent HF visit with intravenous diuretic treatment in the past 12 months	mineralocorticoid antagonists, angiotensin-converting enzyme inhibitor, angiotensin II receptor blocker or angiotensin receptor neurolysin inhibitor, thiazide diuretics, potassium-sparing diuretics
NA	Ovchinnikov AG, et al. (22)	Russia	HFpEF-Specific trial	60 (30 vs. 30)	HFpEF with T2D	HFpEF >50%	66 ± 7 vs. 67 ± 7	13 (43) vs. 10 (33)	I: 0 vs. 0 II: 24 (80) vs. 19 (63) III: 6 (20) vs. 11 (37) IV: 0 vs. 0	59 ± 8 vs. 61 ± 7	all patients were diabetic	empagliflozin 10 mg once daily vs. previously taken hypoglycemic therapy (control group)	24 weeks	NR	ACE/ARB statins DDP4-Inhibitors loop diuretics spironolactone calcium antagonist insulin
The DELIVER trial	Solomon Scott D. et al. (9)	USA	HFpEF-Specific trial	6,263 (3,131 vs. 3,132)	chronic heart failure and a left ventricular ejection fraction of more than 40%	EF ≥40%	71.8 ± 9.6 vs. 71.5 ± 9.5	1,767 (56.4) vs. 1,749 (55.8)	II:2,314 (73.9) vs. 2,399 (76.6) III:807 (25.8) vs. 724 (23.1) IV:10 (0.3) vs. 8 (0.3)	54.0 ± 8.6 vs. 54.3 ± 8.9	1,401 (44.7) vs. 1,405 (44.9)	dapagliflozin at a dose of 10 mg daily vs. matching placebo,	2.3 years	Patients may be ambulatory, or hospitalized; patients must be off intravenous heart failure therapy (including diuretics) for at least 12 h prior to enrollment and 24 h prior to randomization.	usual therapy
The CANDLE trial	Tanaka A., et al. (24)	Japan	Subgroup analysis	165(Canagliflozin = 78 vs. Glimepiride = 87)	Patients with T2D and stable CHF (HFpEF and HFrEF)	HFrEF <50% HFpEF ≥50%	68.3 ± 9.8 VS 68.9 ± 10.4	88 (77.9) vs. 86 (71.7)	I:72 (63.7) vs. 76 (63.9) II:39 (34.5) vs. 40 (33.6) III:2 (1.8) vs. 3 (2.5) unknown:0 (0.0) vs. 1	LVEF <50%: 35 (31.0) vs. 33 (27.5)	all patients were diabetic	canagliflozin 100 mg once daily or glimepiride 0.5 mg once daily	24 weeks	NA	ACE inhibitor or ARB Beta-blocker Calcium channel blocker MRA Diuretic Digitalis Statin Anti-platelet or anti-coagulant Diabetic Insulin Metformin Alpha-glucosidase inhibitor DPP-4 inhibitor GLP-1RA
The DECLARE–TIMI 58	Wiviott Stephen D., et al. (26)	USA	Subgroup analysis	17,160 (8,582 vs. 8,578)	type 2 diabetes who had or were at risk for atherosclerotic cardiovascular disease	HFpEF >45%	63.9 ± 6.8 vs. 64.0 ± 6.8	5,381 (63.1) vs. 5,327 (62.1)	N.R.	N.R.	all patients were diabetic	10 mg of dapagliflozin daily or matching placebo	4.2 years	NA	other glucose-lowering agents by physician discretion
CHIEF-HF	Spertus J. A., et al. (23)	USA	Subgroup analysis	267 (132 vs. 135)	HFpEF with or without diabetes	HFpEF >40%	N.R. separately for HFpEF patients	N.R. separately for HFpEF patients	N.R. separately for HFpEF patients	N.R. separately for HFpEF patients	N.R. separately for HFpEF patients	canagliflozin 100 mg once daily vs. placebo	12 weeks	NA	Participants should be receiving guideline recommended HF medications as prescribed by their treating physician(s) [such as ACEi, ARB, beta-adrenergic blocking agent or beta blocker (*β*blocker), oral diuretics, MRA, angiotensin receptor-neurolysin inhibitor]
CANONICAL	Ueda T., et al. (25)	Japan	HFpEF-Specific trial	82 (42 vs. 40)	HFpEF with T2D	HFpEF ≥50%	76.5 ± 6.4 vs. 75.9 ± 5.8	28 (66.7) vs. 27 (67.5)	I: 0 vs. 0 II: 37 (88.1) vs. 38 (95) III: 5 (11.9) vs. 2 (5) IV: 0 vs. 0	61.1 ± 7.8 vs. 61.9 ± 7.6	all patients were diabetic	canagliflozin 100 mg once daily vs. standard therapy	24 weeks	NYHA cardiac function classification of II–III in the 8 weeks prior to the date of informed consent	ongoing diabetes treatment (GLP-1 receptor agonist, DPP-4, Sulfonylurea, Metformin, Insulin) and cardiovascular drugs (Diuretics, Statin or ezetimibe, Beta-blocker, ACE inhibitor or ARB, Antiplatelet agents)
VERTIS CV	Cosentino F., et al. (18)	USA	Subgroup analysis	1,007 (680 vs. 327)	HFmrEF or HFpEF T2D ASCVD	HFpEF >45% (only some patients in this study are included and there is no classification for HFrEF or HFpEF or HFmrEF)	63.8 vs. 64.7 (mean)	446 (65.6) vs. 207 (63.3)	I: 22.5 vs. 25.7 II: 67.1 vs. 67.6 III: 7.1 vs. 4.6 IV: 0.1 vs. 0	N.R.	all patients were diabetic	ertugliflozin 5 mg once daily vs. ertugliflozin 15 mg once daily vs. placebo	mean of 3.5 years (median 3.0 years)	NA	diuretics, MRAs, β-blockers, ACEI/ARBs, antiplatelets, and statins background standard of care diabetes therapy
EMPEROR-Preserved2	Filippatos G., et al. (20)	Greece	Subgroup analysis	5,988 (2,997 vs. 2,991)	HFpEF with or without T2D	HFpEF >40%	71.8 ± 9.3 vs. 71.9 ± 9.6	1,659 (55.4) vs. 1,653 (55.3)	I: 3 (0.1) vs. 1 (<0.1) II: 2,432 (81.1) vs. 2,451 (81.9) III: 552 (18.4) vs. 531 (17.8) IV: 10 (0.3) vs. 8 (0.3)	54.3 ± 8.8 vs. 54.3 ± 8.8	1,466 (48.9) vs. 1,472 (49.2)	empagliflozin 10 mg daily vs. placebo	52 weeks	NA	ACE inhibitor, ARB, ARNi, Diuretic other than MRA, β-blocker, Lipid-lowering, Aspirin, Anticoagulants, Biguanide, Insulin, Sulfonylurea, DPP-4 inhibitors, GLP-1 receptor analogues
EXCEED	Akasaka H., et al. (14)	Japan	HFpEF-Specific trial	68 (36 vs. 32)	T2D with HFpEF	HFpEF ≥50%	71.9 ± 8.0 vs. 70.3 ± 8.5	22 (61.1%) vs. (19) 59.4%	I:30 (83.3) vs. 28 (87.5) II:6 (16.7) vs. 4 (12.5) no patients in class III or IV	60.9 ± 7.0 vs. 60.4 ± 8.2	all patients were diabetic	ipragliflozin vs. conventional treatment (dosage not reported)	24 weeks	NR	conventional therapy

HF, heart failure; CHF, chronic heart failure; HFrEF, heart failure with reduced ejection fraction; HFmrEF, heart failure with moderate ejection fraction; HFpEF, heart failure with preserved ejection fraction; T2D, type 2 diabetes; NYHA, The New York Heart Association. Classification; LVEF, left ventricular ejection fraction; CKD, chronic kidney disease; ASCVD, atherosclerotic cardiovascular disease .

### Risk of bias in included studies

Overall, nine studies had a low risk of bias, six had some concerns (primarily due to issues in the domain of randomization), and none were at high risk (Supplementary Figure 1).

### Results of the meta-analysis

#### Primary composite outcome (cardiovascular death and hospitalization/urgent visit)

**Figure 2:**
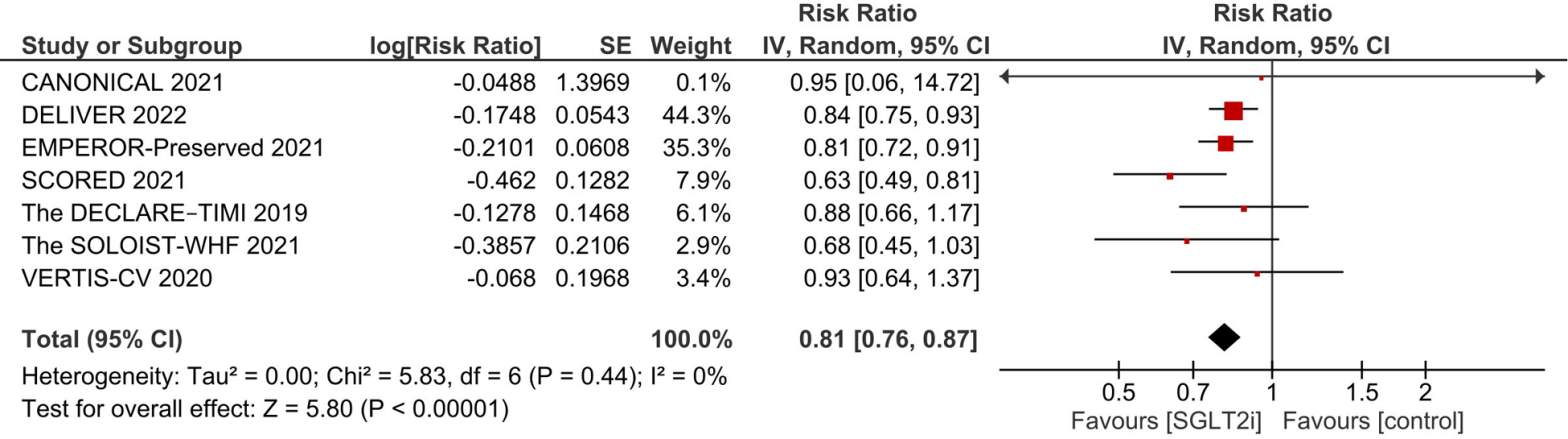
Effect of SGLT2i on the primary composite endpoint of incidence of first HF hospitalization and cardiovascular death.

After pooling the results of 7 studies (9, 15–18, 25, 26), a significant reduction in the incidence of primary composite outcome was observed in the SGLT2i group (RR 0.81, 95% CI: 0.76, 0.87; *I*^2^ = 0%; Figure 2).

#### Cardiovascular death

**Figure 3:**
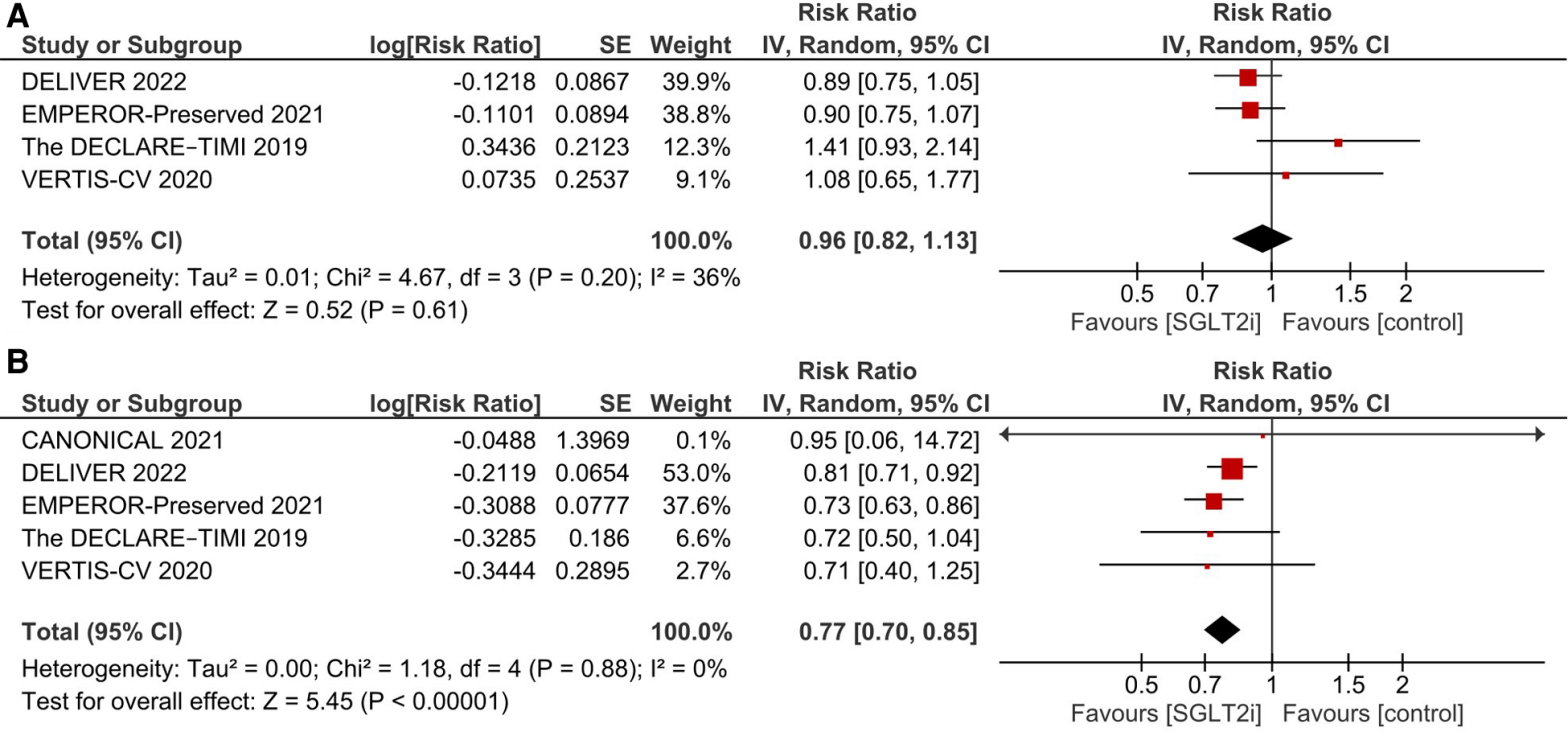
Effect of SGLT2i on: (**A**) cardiovascular death; and (**B**) first HF hospitalization.

There was no significant difference between the SGLT2i and the control groups regarding cardiovascular death (RR 0.96, 95% CI: 0.82, 1.13; *I*^2^ = 36%; Figure 3).

#### First HF hospitalization

SGLT2i were associated with a significant reduction in the incidence of first HF hospitalization (RR 0.77, 95% CI: 0.70, 0.85; *I*^2^ = 0%; Figure 3).

#### All-cause mortality

There was no significant difference in all-cause mortality between the two groups (RR 0.97, 95% CI: 0.89, 1.05; *I*^2^ = 0%) (Supplementary Figure 2).

#### Quality of life

Several questionnaires were used to assess the patient's quality of life including the KCCQ and MLHF scales. Overall, there was a significant improvement in the quality of life in the SGLT2i group (SMD 0.13, 95% CI: 0.06, 0.20; *I*^2^ = 51%; Supplementary Figure 3).

#### Safety

There was no significant difference in the incidence of AEs between the two groups (RR 0.96, 95% CI: 0.86, 1.08; *I*^2^ = 22%; Supplementary Figure 4). Furthermore, no significant difference was observed in the risk of SAEs (RR 0.94, 95% CI: 0.86, 1.02; *I*^2^ = 46%; Supplementary Figure 5). Our analyses regarding specific AEs of interest showed a significant increase in the rate of hypotension and urinary tract infection (Supplementary Table 3).

### Subgroup analyses

#### Diabetes

There was no significant change in the effects of the SGLT2i on the primary composite outcome (cardiovascular death and hospitalization) (*P_interaction_* = 0.91; Supplementary Figure 6), cardiovascular death (*P_interaction_* = 0.09; Supplementary Figure 7), and hospitalization (*P_interaction_* = 0.78; Supplementary Figure 8) due to diabetes.

#### EF diagnostic threshold

There were no between-group differences (EF >40%, >45% or >50%) in the primary composite outcome (*P_interaction_* = 0.57; Supplementary Figure 9), and risk of hospitalization (*P_interaction_* = 0.88; Supplementary Figure 10). Regarding cardiovascular death, there was a trend towards greater benefit with SGLT2i use in studies with EF >40% as the cutoff (RR 0.89, 95% CI: 0.79, 1.01; Supplementary Figure 1^1^) as compared with studies with EF >45% which showed a non-significant increase in cardiovascular mortality (RR 1.26, 95% CI: 0.92, 1.74; *P_interaction_* = 0.05).

#### Baseline LVEF

The results of our primary analysis for the primary composite outcome were consistent across the HFmrEF (EF between 40% and 50%) and HFpEF patient cohorts (EF more than 50%) (*P_interaction _*= 0.48; Supplementary Figure 1^2^).

#### Type of study

The results of all three primary outcomes were consistent across the type of study (HFpEF-specific trials vs. subgroup/posthoc analysis studies) (Supplementary Figures 13–S15).

#### Certainty of evidence

The summary of findings and quality of evidence for study outcomes is available in Table 2. The certainty of evidence was high for all outcomes except for the quality of life which was downgraded to moderate due to the issue of indirectness.

**Table 2:** GRADE summary of findings.

Outcome	No. of studies	Effect estimate (95% CI)	Risk of bias	Inconsistency	Indirectness	Imprecision	Quality of evidence (GRADE)
Primary composite outcome (cardiovascular death and hospitalization)	7	RR 0.81 [0.76, 0.87]	Not serious	Not serious	Not serious	Not serious	High
Cardiovascular death	4	RR 0.96 [0.82, 1.13]	Not serious	Not serious	Not serious	Not serious	High
Hospitalization	5	RR 0.77 [0.70, 0.85]	Not serious	Not serious	Not serious	Not serious	High
All-cause mortality	8	RR 0.97 [0.89, 1.05]	Not serious	Not serious	Not serious	Not serious	High
Quality of life	7	SMD 0.13 [0.07, 0.20]	Not serious	Not serious	Serious	Not serious	Moderate
Any adverse events	5	RR 0.96 [0.86, 1.08]	Not serious	Not serious	Not serious	Not serious	High
Serious adverse events	6	RR 0.94 [0.86, 1.02]	Not serious	Not serious	Not serious	Not serious	High

## Discussion

To the best of our knowledge, this is the most comprehensive systematic review and meta-analysis to date investigating the effect of SGLT2i on the clinical outcomes of patients with HFpEF and HFmrEF. Based on our analyses, SGLT2i significantly decrease the incidence of primary composite outcome (cardiovascular death and first HF hospitalization) mainly driven by the decrease in hospitalization, and substantially improve the quality of life. In addition, our subgroup analyses did not show any significant between-group differences in most of the outcomes assessed.

Overall, the results of our meta-analysis are congruent with the findings of prior meta-analyses, demonstrating a significant benefit of SGLT2i in HFpEF patients (27, 28). We included the DELIVER trial which is the largest RCT specifically conducted for HFpEF and HFmrEF patient population. This enabled us to extend the results of the previous meta-analyses by pooling a significantly greater cumulative sample size. Notably, our finding of no reduction in cardiovascular death is contrary to the results of a recent meta-analysis which found that SGLT2i decreased the risk of cardiovascular death (29). This meta-analysis, however, pooled results only from the EMPEROR-Preserved and DELIVER trials whereas we also included data available from other RCTs. Although this may have introduced some heterogeneity due to the inclusion of non-HF-specific RCTs, it also increases the statistical power required to discern a potential benefit. Nevertheless, further large-scale RCTs are required to resolve this inconsistency and establish the benefit of SGLT2i for reducing cardiovascular mortality with greater confidence. This also indicates that therapies that decrease the risk of mortality in HFpEF/HFmrEF patients are still desperately needed.

A recent meta-analysis reported similar findings to ours but it did not explore the benefit of SGLT2i in improving the quality of life of patients (30). Our study showed that SGLT2i significantly improve the quality of life based on KCCQ and MLHF questionnaires which is an important finding for patients who desire effective treatment options that can not only alleviate symptoms but also improve their overall well-being and day-to-day functioning. Most importantly, since there is a paucity of data regarding the use of SGLT2i in HFmrEF patients, we performed a subgroup analysis based on individual LVEF status and demonstrated that the beneficial effect of SGLT2i is consistent in this population too. Although the overlap of results of trials with HFrEF and HFpEF patients may shed light on the pharmacological treatment of HFmrEF, there is still a lack of specifically designed RCTs for this group of patients (31). More RCTs exclusively designed for this subset of HF patients are required to determine whether or not SGLT2i will reduce the rate of cardiovascular death alone.

The mechanism of action of SGLT2i involves the inhibition of sodium-glucose cotransporter-2 located in the S1 and S2 segments of the proximal convoluted tubule (PCT). Simultaneous prevention of sodium and glucose reabsorption leads to glucosuria and natriuresis (32). However, since the cardiovascular benefits of SGLT2i are demonstrated early after the initiation of therapy, mechanisms of action other than glycemic control seem to be responsible for these effects since improved glycemic control requires years to be effective (33, 34). Several cardioprotective effects including decreased risk of the development and decompensation of HF, reduction in blood pressure, and maintaining proper renal glomerular function are resulted from the diuretic effects along with tissue sodium regulation provided by SGLT2i (35). A recent proteomics study suggests the enhanced autophagy induced by SGLT2i as a potential mechanism underlying the cardioprotective effects (36). Moreover, a metabolomic study stated that alterations in cardiac cell metabolism towards the increased consumption of ketone bodies and free fatty acids may be responsible for these effects (37). Other suggested benefits include prevention of left ventricular hypertrophy, adaptive cellular reprogramming, vascular compliance, reduced blood pressure, reduced systemic inflammation, weight loss, enhanced myocardial energetics, lower uric acid levels, and positive effects on endothelial progenitor cells (38–41).

The use of SGLT2i is associated with several adverse events, including a higher risk of amputations, fractures, bladder cancer, and diabetic ketoacidosis (DKA) (42). When considering specific adverse events, hypotension and urinary tract infection were more frequently seen in the intervention group which aligns with the previously published literature as well-known adverse events of SGLT2i (43, 44).

Our study has several strengths. Our study has the largest cumulative sample size by including comprehensive and up-to-date results. We included only RCTs to review the highest level of clinical evidence. In addition, the GRADE criteria were used for assessing the quality of the evidence. supplementary material from all of the studies was meticulously explored to achieve comprehensive data. Moreover, we performed several subgroup analyses stratified on different EF intervals as well as concurrent diagnoses of DM. Furthermore, the heterogeneity of the studies in our analyses was assessed as very low as demonstrated by the *I*^2^ statistic. Our study is also susceptible to certain limitations. First, some of the data were obtained from the post-hoc analyses as the original studies included HFrEF patients as well. Second, the proportion of patients with DM varied among different studies, with some of the studies not including DM patients. Third, studies differed in terms of EF thresholds attributed to each type of heart failure. Fourth, differences exist in both the type and dosages of SGLT2i used as well as the duration of the studies.

Based on our analysis, the use of SGLT2i is associated with a lower risk of the primary composite outcome of hospitalization and cardiovascular death mainly driven by the reduction in hospitalization, and a higher quality of life among HFpEF/HFmrEF patients. Further research involving longer follow-up periods is required to draw a comprehensive conclusion regarding the efficacy and safety of SGLT2i in HFpEF and HFmrEF patients, especially for cardiovascular death.

## Data Availability

The raw data supporting the conclusions of this article will be made available by the authors, without undue reservation.
